# Epidemiology of neonatal disorders attributable to low birthweight-global burden of disease research, 1990–2021

**DOI:** 10.3389/fped.2024.1461134

**Published:** 2025-01-31

**Authors:** Qi Zhang, Lan Luo, Lai-lai Yan, Jing Guo, Hui-jun Wu, Zi-wei Zhang, Yu-hua Zhu, Rui Qiao

**Affiliations:** ^1^ Department of Public Health, Baotou Medical College, Baotou, China; ^2^ Department of Laboratorial Science and Technology, School of Public Health, Peking University, Beijing, China; ^3^ Key Laboratory of Epidemiology of Major Diseases, Peking University, Ministry of Education, Beijing, China; ^4^ Department of Gynecology, Kundulun District Hospital, Baotou, China; ^5^ Department of Gynecology and Obstetrics, Sinopharm Northern Hospital, Baotou, China; ^6^ Department of Public Health, International College, Krirk University, Bangkok, Thailand

**Keywords:** global health, epidemiology, low birthweight, neonatal disease burden, health inequality

## Abstract

**Background:**

To evaluate global, regional, and national trends in the burden of neonatal diseases attributable to LBW, as well as associated health inequalities, from 1990 to 2021.

**Methods:**

Using data from the Global Burden of Disease Study (GBD2021), we analyzed deaths and DALYs due to LBW-attributable neonatal diseases. Data were stratified by gender, geographic region, epidemiological characteristics, and SDI levels. Trends and influencing factors were investigated through Joinpoint regression, health inequality analysis, and frontier modeling.

**Results:**

In 2021, the global age-standardized mortality rate (ASMR) and age-standardized disability-adjusted life year (DALY) rate (ASDR) for neonatal diseases associated with low birth weight (LBW) were 22.76 [95% uncertainty interval (UI): 19.63–26.40] and 2,227.54 (95% UI: 1,939.96–2,563.52) per 100,000, respectively. Over the past 32 years, these rates have consistently declined, with average annual percentage changes (AAPCs) of −1.40 [95% confidence interval (CI): −1.48 to −1.33] for ASMR and −1.27 (95% CI: −1.34 to −1.21) for ASDR. Notably, absolute health inequality related to the Socio-demographic Index (SDI) has decreased, as indicated by a reduction in DALY disparities between the most and least developed countries from −4,216.49 (95% CI: −4,558.27 to −3,874.71) in 1990 to −2,635.35 (95% CI: −2,868.40 to −2,402.30) in 2021. However, relative health inequality has worsened, with the relative disease burden in low-SDI countries increasing from −33.46% (95% CI: −36.29% to −30.63%) to −40.20% (95% CI: −44.02% to −36.39%). The burden of neonatal diseases remains disproportionately concentrated in low-SDI regions. Frontier analyses highlight opportunities for improvement across development levels. Some low-SDI countries have achieved minimal theoretical disease burdens, whereas certain high-SDI countries lag in reducing their neonatal disease burdens.

**Conclusion:**

Over the past 32 years, the global burden of neonatal diseases attributable to LBW has significantly decreased, but inequality in disease burden has intensified. Addressing this disparity requires sustained international and governmental efforts to improve the accessibility, equality, and quality of healthcare for pregnant women and newborns.

## 1 Introduction

The global decline in total fertility rates has amplified the prominence of neonatal health as a critical public health concern (1). The United Nations Sustainable Development Goals (SDG 3.2) emphasize eliminating preventable deaths among newborns and children under five years of age by 2030 (2). Despite substantial progress in neonatal and child health over the past three decades, projections indicate that over 30% of countries are unlikely to meet these goals by 2030 under current conditions (3). In 2021, there were 27.06 million cases of neonatal diseases worldwide, making them the third largest contributor to disability-adjusted life years (DALYs) across all age groups globally (4). Reducing the burden of neonatal diseases is thus a pressing global health priority and a key challenge in achieving the Sustainable Development Goals.

Low birthweight (LBW), defined as live-born infants weighing less than 2,500 g, is among the three major risk factors for neonatal diseases, alongside preterm birth and exposure to PM2.5 pollution (5). According to the World Health Organization (WHO), approximately 15% of live-born infants globally have LBW (6). This metric serves as a critical indicator of neonatal health, given its strong association with elevated mortality rates and numerous neonatal complications, including neurological, respiratory, and cardiovascular diseases (7–10). Recent studies have also linked LBW to long-term health consequences, including heightened vulnerability to severe COVID-19 in adulthood (11). Furthermore, LBW is the leading risk factor for the childhood disease burden, accounting for 6.3% of total global DALYs (12). While prior research has explored trends in neonatal disease burden and LBW prevalence (13, 14), there has been limited analysis of the neonatal disease burden specifically attributable to LBW and the associated health inequalities at global, regional, and national levels. This study addresses this gap by leveraging Global Burden of Disease (GBD) 2021 data to assess the spatiotemporal trends of neonatal disease burden attributable to LBW and associated health inequalities from 1990 to 2021. The findings aim to inform health policymakers in developing targeted maternal and neonatal health policies, enhancing the accessibility and quality of perinatal care, improving neonatal health outcomes, and promoting health equality.

## 2 Methods

### 2.1 Data sources

The GBD 2021 study is the most comprehensive ecological epidemiological study to date, systematically analyzing disease burden data for 371 diseases and injuries and 88 risk factors across 204 countries from 1990 to 2021. Utilizing Bayesian meta-regression and spatiotemporal Gaussian process regression mixed-effects models, it provides robust estimates (4). For this study, we extracted data from the Global Health Data Exchange website (https://vizhub.healthdata.org/gbd-results/) for secondary analysis. Variables included deaths, disability-adjusted life years (DALYs), age-standardized mortality rates (ASMR per 100,000), ASDR (per 100,000), and their 95% uncertainty intervals (UI) for neonatal diseases attributable to LBW from 1990 to 2021. Countries were categorized into 21 GBD regions based on epidemiological and geographical factors. Age-standardized metrics were calculated using the GBD world standard population, with detailed methodology available in prior publications (4). Countries were further classified into five SDI categories—low, low-middle, middle, high-middle, and high SDI—based on fertility rates under age 25, mean years of education, and per capita income, with SDI values ranging from 0 (lowest socioeconomic development) to 1 (highest) (15).

### 2.2 Diseases and risk factors

Neonatal diseases in the GBD 2021 study are categorized into five groups based on ICD-10: neonatal preterm birth, neonatal encephalopathy due to birth asphyxia and trauma, neonatal sepsis and infections, hemolytic disease and jaundice, and other neonatal conditions. Unlike the traditional 2,500-g cutoff, the GBD defines LBW as any birth weight below the theoretical minimum risk exposure level (TMREL). Given the strong association between LBW and short gestation, the GBD quantified the combined attributable burden using joint exposure to LBW and short gestation, relative risk, and population attributable fraction (PAF). It then separated the independent PAF due to LBW to quantify the disease burden adjusted for gestational age. LBW is defined as any 500-gr unit below the TMREL, identified as [38, 40) weeks of gestation and [3,500, 4,000) grams. This approach effectively distinguishes LBW from preterm birth. Detailed calculation and modeling methods are described in previous literature (4).

### 2.3 Statistical analysis

To account for population age structure variations across regions, we used age-standardized mortality rates (ASMR) and age-standardized disability-adjusted life year rates (ASDR) to assess the burden of neonatal diseases attributable to LBW. Trends were analyzed using the Joinpoint regression model to calculate annual percentage changes (APCs) and average annual percentage changes (AAPCs) with 95% confidence intervals (CI), identifying potential inflection points. APCs highlight linear trends over specific periods, while AAPCs evaluate overall trends across the study period. The calculation methodology is as follows:

$$\begin{array}{rl} & \mathrm{In}\;(\mathrm{ASMR}\;\mathrm{or}\;\mathrm{ASDR})\;=\;\alpha \;+\;\beta_{i}x\\ & \mathrm{APC}s_{i}\;=\;\{\exp(\beta_{i})-1\}\;\times 100\\ & \mathrm{AAPCs}\;=\;\left\{\exp\left(\sum w_{i}\beta_{i}/\sum w_{i}\right)-1\right\}\;\times 100 \end{array}$$

In this equation, *x* denotes the year, *β_i_* is the slope coefficient for each segment within defined intervals, and *w_i_* represents the time range for each segment. When the average annual percentage change (AAPC) is not equal to 0, the change is considered statistically significant. If 0 lies within the 95% confidence interval (CI) of the AAPC, the trend is deemed not statistically significant. Conversely, a 95% CI with both limits positive indicates an increasing trend, while both limits being negative indicates a decreasing trend (16).

To assess health inequality in the burden of neonatal diseases attributable to low birth weight (LBW) across countries grouped by Socio-Demographic Index (SDI) levels, we adopted methods recommended by the World Health Organization. Absolute health inequality was quantified using the slope index of inequality (SII), while relative health inequality was measured with the concentration index (CI) (17). Specifically, countries were ranked by SDI levels, with their relative order based on the median cumulative population fraction. The SII reflects the slope of the regression line between age-standardized death rates (ASDR) and relative order, with a positive SII indicating a higher disease burden in high-SDI countries, and a negative SII indicating the opposite. The CI, calculated as the area between the Lorenz curve [constructed by fitting disability-adjusted life years (DALYs) to cumulative population] and the diagonal (line of absolute equality), represents the distribution of disease burden. A negative CI value indicates concentration of disease burden in low-SDI countries, while a positive CI indicates concentration in high-SDI countries. The closer the absolute CI value is to 0, the greater the equality; the closer it is to 1, the lower the equality (18).

To further evaluate the efficiency and potential of countries in mitigating the burden of neonatal diseases attributable to LBW, we developed a stochastic frontier model. This model quantitatively assesses the gap between a country's actual disease burden and its theoretical minimum burden (frontier level) based on its SDI. The frontier line represents the minimum achievable ASDR for neonatal diseases under optimal conditions for a given SDI level. The gap between a country's observed ASDR and this frontier line is termed the efficiency difference, reflecting the disparity between theoretical minimum and observed burden. Using data from 1990 to 2021, we employed data envelopment analysis to plot the ASDR frontier for neonatal diseases attributable to LBW. To incorporate uncertainty, a resampling method with 1,000 iterations was applied, where countries were randomly sampled and replaced across all years. The mean ASDR for each SDI value in the resampled data was calculated, and a polynomial regression with a degree of freedom of 1 and smoothness of 2 was used to plot the boundary. For 2021, we quantified the efficiency difference by calculating the distance between each country's ASDR and the frontier line. Countries with ASDR below the frontier were assigned an efficiency difference of 0 and categorized as frontier countries (19).

Data cleaning, analysis, and visualization in this study were conducted using R software (version 4.3.3). Joinpoint regression analysis was performed with the Joinpoint Regression Program (Command-Line version 5.2.0), while the relative health inequality index (CI) was calculated using STATA 16.0.

## 3 Results

### 3.1 Global

**Figure 1:**
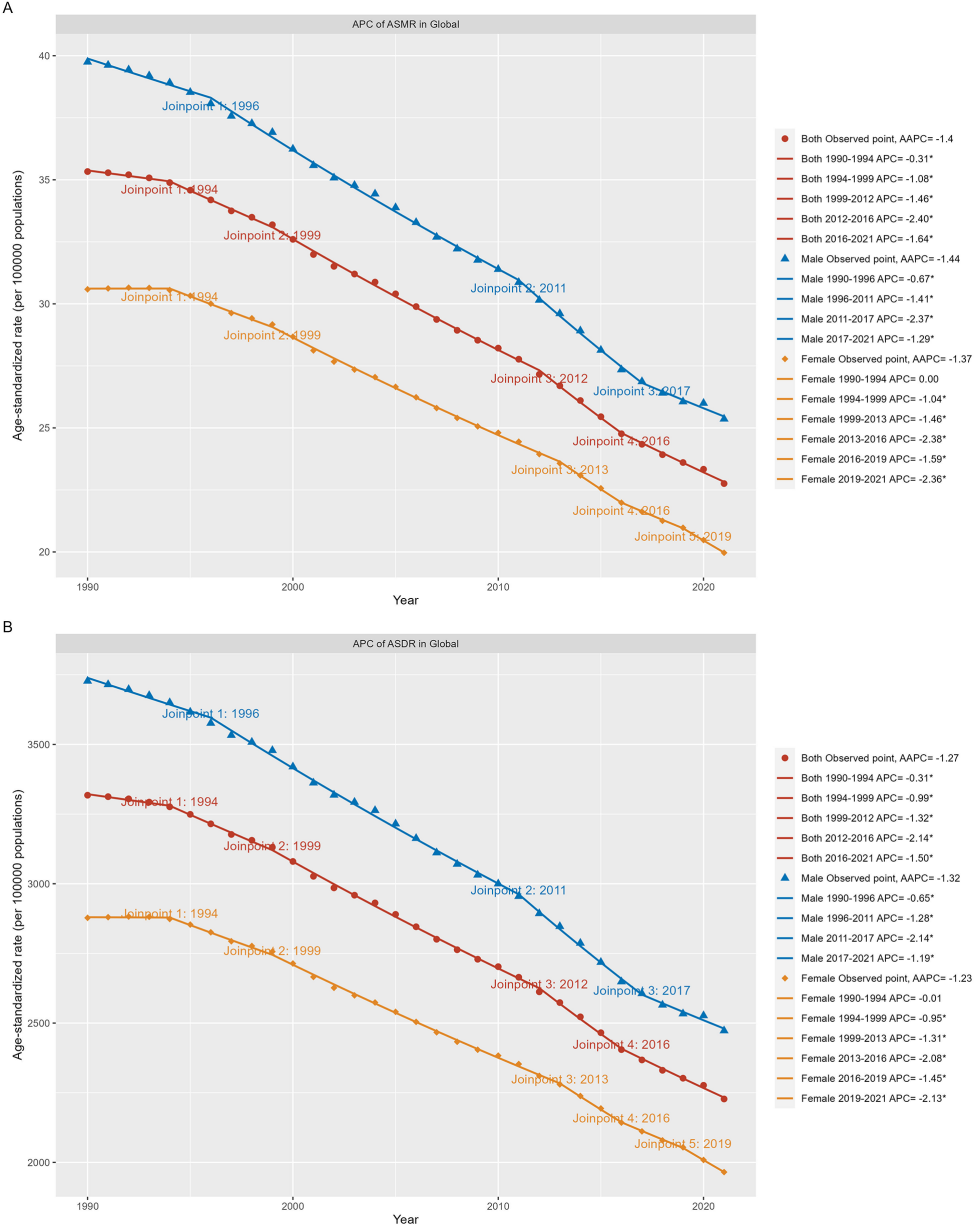
Global trends for ASR (per 100,000 population) of the burden of neonatal diseases attributable to LBW from 1990 to 2021. **(A)** ASMR by sex globally. **(B)** ASDR by sex globally. LBW, low birthweight; ASMR, age-standardized rate of mortality rate; ASDR, age-standardized disability-adjusted life-year rate. *P < 0.05.

According to GBD 2021 estimates, age-standardized mortality rates (ASMR) and disability-adjusted life years (ASDR) for neonatal diseases attributable to LBW have both declined globally from 1990 to 2021. In 2021, the global ASMR was 22.76 (95% UI: 19.63–26.40) per 100,000 live births, with an average annual percentage change (AAPC) of −1.40 (95% CI: −1.48 to −1.33). Similarly, the global ASDR was 2,227.54 (95% UI: 1,939.96–2,563.52) per 100,000 live births, with an AAPC of −1.27 (95% CI: −1.34 to −1.21). The burden, measured by both ASMR and ASDR, was consistently higher in males than females (Table 1). The Joinpoint regression model revealed a steady decline in ASMR and ASDR over the 32-year period, with the most rapid reductions occurring from 2012 to 2016 (Figures 1A,B). Notably, between 2019 and 2021, females experienced the fastest decline in ASDR (Figure 1B).

**Table 1:** Characteristics and trends of ASMR and ASDR of neonatal disease burden attributable to low birthweight globally and in SDI regions and GBD regions from 1990 to 2021.

Characteristics	ASMR (95% UI)			ASDR (95% UI)		
	1990	2021	AAPCs	1990	2021	AAPCs
Global	35.33 (33.37, 37.50)	22.76 (19.63, 26.4)	−1.40 (−1.48, −1.33)*	3,317.75 (3,135.34, 3,527.04)	2,227.54 (1,939.96, 2,563.52)	−1.27 (−1.34, −1.21)*
Sex						
Male	39.75 (37.04, 42.64)	25.36 (21.56, 29.89)	−1.44 (−1.52, −1.36)*	3,727.56 (3,492.68, 3,988.37)	2,472.64 (2,125.59, 2,897.5)	−1.32 (−1.39, −1.24)*
Female	30.59 (28.67, 32.72)	19.97 (17.32, 22.82)	−1.37 (−1.48, −1.26)*	2,877.67 (2,686.29, 3,076.09)	1,965.13 (1,711.55, 2,231.66)	−1.23 (−1.33, −1.12)*
SDI regions						
High SDI	7.18 (6.88, 7.50)	3.02 (2.70, 3.32)	−2.75 (−2.94, −2.57)*	765.34 (725.31, 812.59)	392.10 (350.03, 435.11)	−2.13 (−2.28, −1.97)*
High-middle SDI	16.01 (14.65, 17.47)	4.23 (3.72, 4.77)	−4.22 (−4.55, −3.9)*	1,536.34 (1,415.27, 1,680.76)	482.13 (429.84, 538.63)	−3.68 (−3.94, −3.42)*
Middle SDI	26.76 (24.97, 28.6)	12.37 (10.67, 14.4)	−2.46 (−2.59, −2.33)*	2,524.09 (2,351.39, 2,705.12)	1,274.50 (1,102.94, 1,466.91)	−2.18 (−2.29, −2.08)*
Low-middle SDI	52.66 (49.35, 56.41)	30.13 (25.86, 35.19)	−1.78 (−1.86, −1.69)*	4,953.55 (4,647.65, 5,300.45)	2,983.76 (2,589.67, 3,440.51)	−1.61 (−1.67, −1.55)*
Low SDI	53.49 (50.18, 57.1)	35.82 (30.1, 42.65)	−1.29 (−1.32, −1.26)*	4,943.41 (4,639.25, 5,286.00)	3,394.35 (2,877.98, 4,006.16)	−1.21 (−1.24, −1.18)*
21 GBD regions						
High-income Asia Pacific	4.19 (3.80, 4.63)	0.97 (0.88, 1.08)	−4.59 (−5.16, −4.02)*	455.42 (413.87, 503.72)	168.93 (142.50, 194.6)	−3.13 (−3.51, −2.75)*
High-income North America	7.25 (7.05, 7.43)	4.23 (3.77, 4.7)	−1.70 (−2.07, −1.32)*	806.32 (757.96, 859.16)	530.20 (471.22, 588.76)	−1.32 (−1.61, −1.02)*
Australasia	5.90 (5.6, 6.21)	2.35 (1.99, 2.78)	−2.83 (−3.58, −2.07)*	675.26 (623.94, 730.67)	335.51 (288.12, 390.62)	−2.16 (−2.71, −1.61)*
Andean Latin America	25.47 (23.23, 28.34)	8.40 (6.59, 10.36)	−3.49 (−3.85, −3.13)*	2,424.89 (2,210.03, 2,687.14)	862.45 (697.31, 1,041.51)	−3.25 (−3.6, −2.9)*
Tropical Latin America	31.08 (27.87, 34.15)	8.88 (7.14, 11.07)	−3.99 (−4.09, −3.88)*	2,881.66 (2,601.09, 3,162.88)	953.56 (784.96, 1,144.17)	−3.54 (−3.68, −3.39)*
Central Latin America	22.80 (21.36, 24.48)	9.28 (7.37, 11.73)	−2.84 (−3.01, −2.67)*	2,150.73 (2,025.03, 2,305.82)	946.32 (774.37, 1,166.73)	−2.57 (−2.81, −2.33)*
Southern Latin America	17.57 (16.84, 18.28)	6.22 (4.89, 7.81)	−3.17 (−3.89, −2.44)*	1,700.95 (1,632.18, 1,779.25)	688.72 (569.58, 827.73)	−2.77 (−3.40, −2.14)*
Caribbean	27.13 (24.42, 30.61)	21.97 (17.75, 26.46)	−0.73 (−0.84, −0.62)*	2,646.35 (2,397.92, 2,968.05)	2,147.81 (1,769.26, 2,554.55)	−0.72 (−0.82, −0.62)*
Western Europe	5.67 (5.52, 5.8)	2.42 (2.13, 2.72)	−2.73 (−2.95, −2.51)*	613.91 (581.74, 651.71)	323.33 (282.23, 362.16)	−2.06 (−2.25, −1.87)*
Central Europe	13.27 (12.68, 13.88)	3.26 (2.76, 3.78)	−4.37 (−4.99, −3.75)*	1,304.04 (1,245.45, 1,370.51)	399.04 (347.50, 453.14)	−3.62 (−4.1, −3.13)*
Eastern Europe	11.26 (10.69, 11.82)	3.17 (2.85, 3.52)	−4.33 (−5.07, −3.57)*	1,083.18 (1,027.93, 1,135.97)	360.01 (328.44, 396.63)	−3.79 (−4.47, −3.1)*
Central Asia	16.26 (15.03, 17.49)	11.06 (9.32, 13)	−1.26 (−1.55, −0.97)*	1,555.53 (1,440.58, 1,674.47)	1,078.61 (921.94, 1,254.72)	−1.20 (−1.48, −0.93)*
North Africa and Middle East	38.81 (35.14, 43.66)	12.24 (10.48, 14.33)	−3.68 (−3.81, −3.55)*	3,661.11 (3,338.94, 4,106.79)	1,253.53 (1,082.88, 1,440.16)	−3.42 (−3.53, −3.31)*
South Asia	59.70 (55.11, 65)	35.43 (29.83, 42.02)	−1.66 (−1.77, −1.55)*	5,655.65 (5,221.16, 6,123.98)	3,584.81 (3,092.96, 4,209.78)	−1.46 (−1.56, −1.37)*
Southeast Asia	25.61 (23.62, 27.64)	12.93 (10.84, 15.16)	−2.20 (−2.30, −2.10)*	2,418.89 (2,231.16, 2,610.5)	1,283.24 (1,098.00, 1,478.69)	−2.04 (−2.13, −1.95)*
East Asia	16.95 (14.53, 19.57)	3.53 (3.01, 4.17)	−4.97 (−5.19, −4.75)*	1,592.65 (1,373.66, 1,819.93)	381.33 (331.70, 440.89)	−4.53 (−4.72, −4.34)*
Oceania	18.41 (15.71, 21.36)	16.95 (13.61, 20.72)	−0.25 (−0.33, −0.18)*	1,810.15 (1,568.69, 2,077.47)	1,663.52 (1,351.07, 2,006.75)	−0.26 (−0.33, −0.19)*
Western Sub-Saharan Africa	56.60 (52.89, 60.89)	42.31 (35.99, 49.29)	−0.94 (−1.00, −0.87)*	5,147.80 (4,806.45, 5,532.45)	3,896.93 (3,333.59, 4,519.7)	−0.89 (−0.95, −0.83)*
Eastern Sub-Saharan Africa	47.26 (43.69, 51)	30.16 (24.56, 37.23)	−1.45 (−1.50, −1.41)*	4,322.03 (3,996.74, 4,662.6)	2,833.52 (2,322.60, 3,469.22)	−1.36 (−1.41, −1.32)*
Central Sub-Saharan Africa	36.90 (32.06, 42.36)	25.33 (20.42, 30.44)	−1.19 (−1.31, −1.08)*	3,372.92 (2,935.75, 3,868.55)	2,354.78 (1,925.37, 2,821.34)	−1.14 (−1.25, −1.02)*
Southern Sub-Saharan Africa	32.78 (29.31, 36.06)	27.70 (22.82, 33.73)	−0.46 (−0.61, −0.31)*	3,040.55 (2,730.72, 3,354.01)	2,611.63 (2,165.64, 3,152.89)	−0.42 (−0.57, −0.27)*

ASMR, age-standardized mortality rate; ASDR, age-standardized disability-adjusted life year rate; AAPC, average annual percentage change.

* *p* < 0.05.

### 3.2 Regions and countries

At the regional level, Western Sub-Saharan Africa recorded the highest ASMR and ASDR in 2021. All 21 GBD regions exhibited a downward trend in ASMR and ASDR over the past three decades, with East Asia experiencing the steepest decline and Oceania the slowest (Table 1).

**Figure 2:**
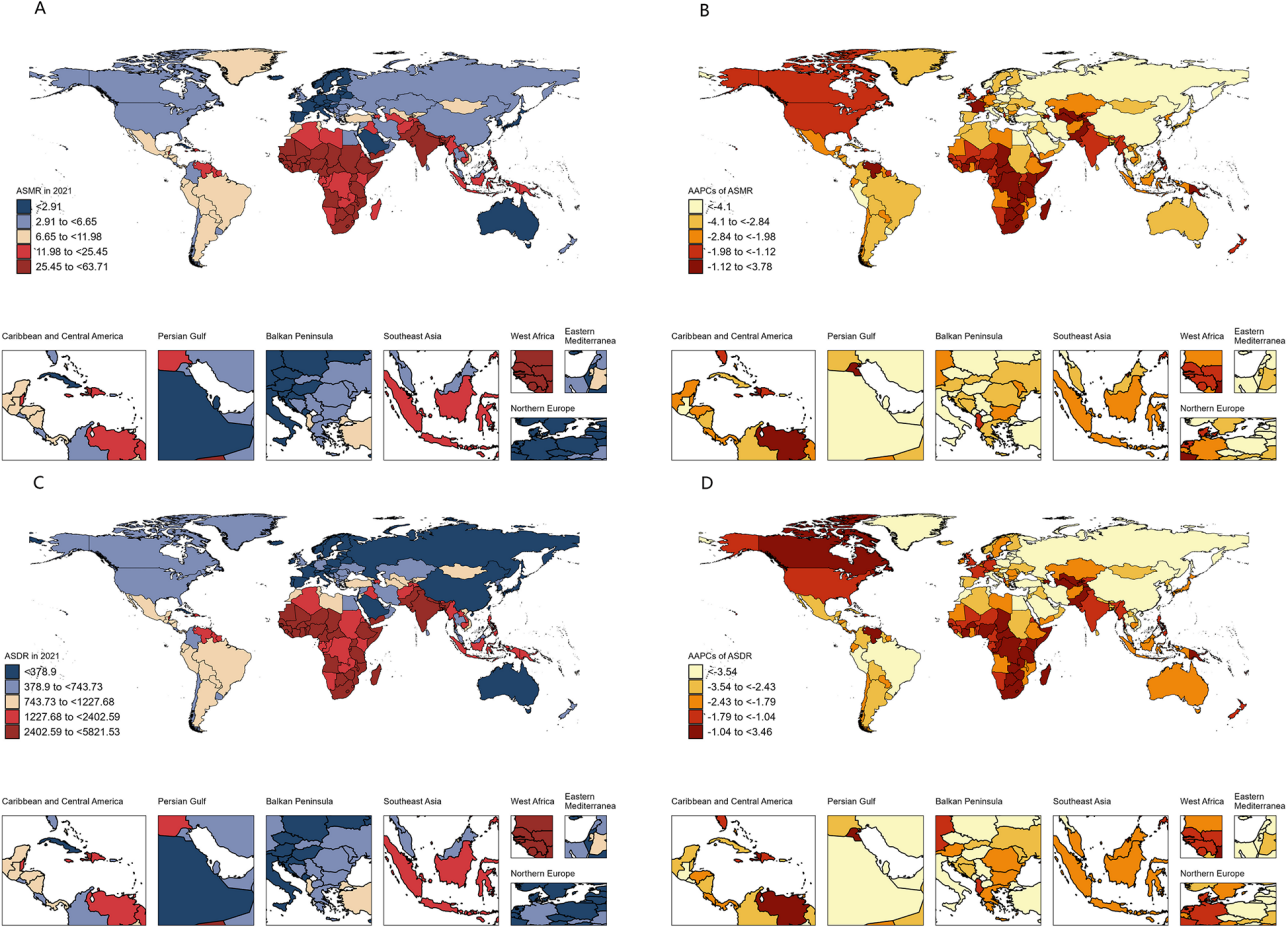
Spatial distribution and changes of the burden of neonatal disorders attributable to LBW in 204 countries and territories. **(A)** ASMR in 2021. **(B)** AAPCs in ASMR. **(C)** ASDR in 2021. **(D)** AAPCs in ASDR. LBW, low birthweight; ASMR, age-standardized rate of mortality rate; ASDR, age-standardized disability-adjusted life-year rate; AAPC, average annual percentage change.

Nationally, among the 204 countries or territories assessed, Mali had the highest ASMR and ASDR in 2021, while Andorra reported the lowest (Figures 2A,C). Compared to 1990, 196 countries showed a decrease in ASMR, with 192 achieving statistically significant reductions. Saudi Arabia demonstrated the most substantial decline (Figure 2B, Supplementary Table S1). For ASDR, 197 countries exhibited a declining trend, with 191 achieving statistically significant reductions. Iran achieved the largest decline, whereas Tokelau recorded the highest growth rates for both ASMR and ASDR (Figures 2B,D, Supplementary Table S1).

### 3.3 Cross-Country health inequality analysis

**Figure 3:**
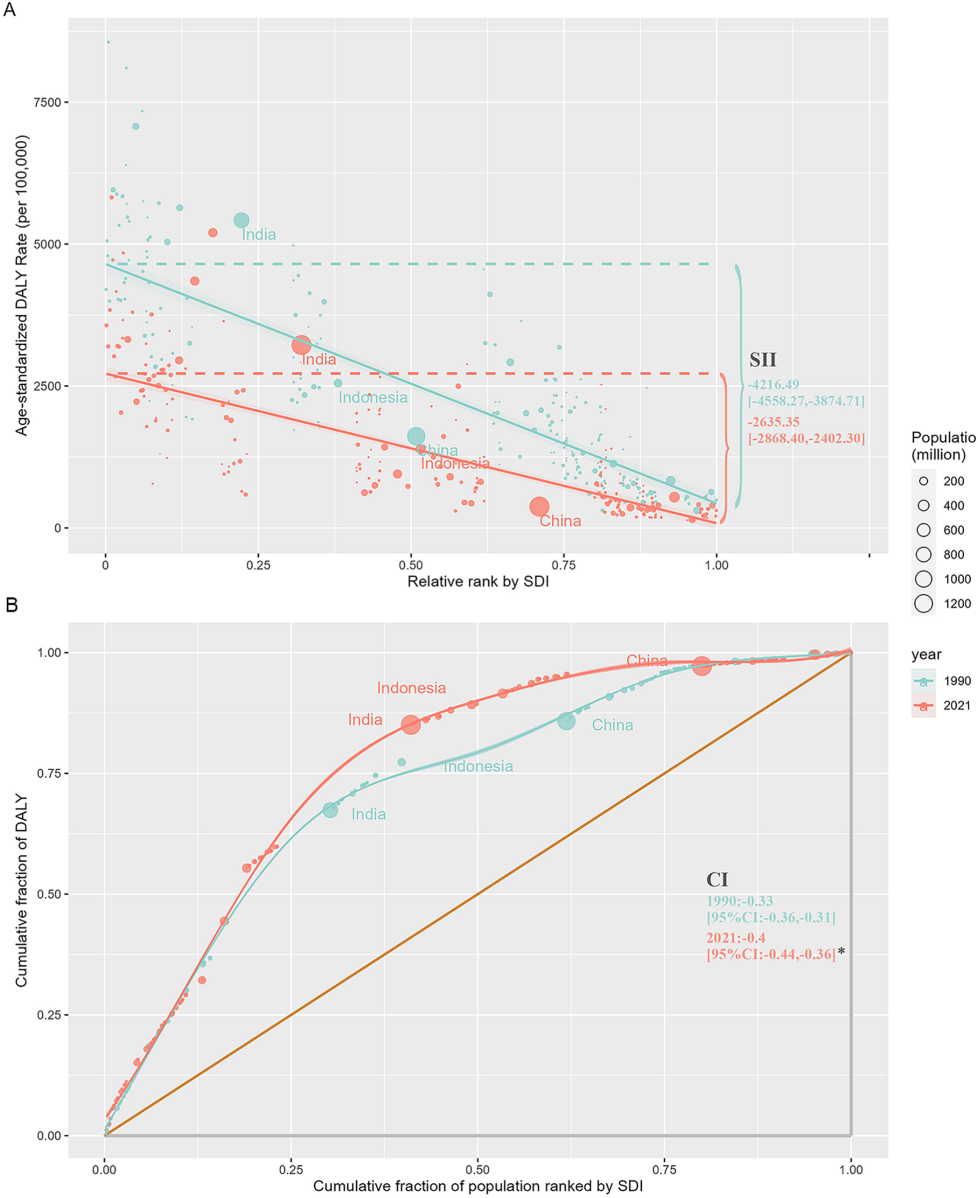
Regression and concentration curves of health inequality related to SDI and ASDR of the burden of neonatal diseases attributable to LBW from 1990 to 2021. **(A)** Inequality regression line. **(B)** Inequality concentration curve. SDI, sociodemographic index; ASDR, age-standardized disability-adjusted life-year rate; LBW, low birthweight; SII, slope index of inequality; CI, concentration index.*P < 0.05.

The analysis also revealed a strong inverse relationship between neonatal disease burden and sociodemographic index (SDI) levels. In 2021, the ASMR and ASDR in low SDI regions were 11.8 and 8.7 times higher, respectively, than in high SDI regions. Between 1990 and 2021, all five SDI regions experienced declining trends in ASMR and ASDR, with the largest decreases observed in the high-middle SDI region (Table 1). Despite this progress, the burden of neonatal diseases attributable to LBW remains disproportionately concentrated in underdeveloped countries. In 2021, India, Nigeria, Pakistan, Ethiopia, and Bangladesh collectively accounted for over 50% of global disability-adjusted life years (DALYs) associated with neonatal diseases, whereas high-income regions in Asia-Pacific and North America represented only 1% of the global total (Figure 2C, Supplementary Table S1). Health inequality analyses underscore these disparities. Between 1990 and 2021, the absolute inequality in disease burden, measured by the slope index of inequality (SII), decreased from −4,216.49 (95% CI: −4,558.27 to −3,874.71) per 100,000 population in 1990 to −2,635.35 (95% CI: −2,868.40 to −2,402.30) in 2021 (Figure 3A). However, the relative inequality, measured by the concentration index (CI), increased from −33.46% (95% CI: −36.29% to −30.63%) in 1990 to −40.20% (95% CI: −44.02% to −36.39%) in 2021, indicating that the disease burden in 2021 was more heavily skewed toward populations in low SDI countries compared to 1990 (Figure 3B).

### 3.4 Frontier analysis

**Figure 4:**
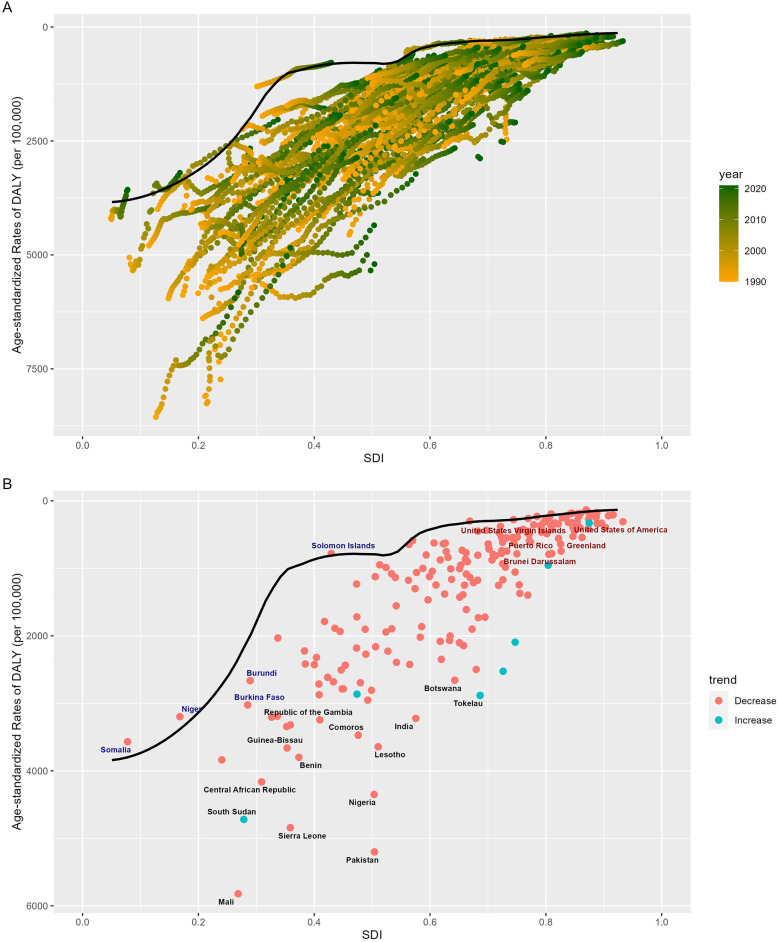
Frontier analysis of SDI and ASDR of the burden of neonatal diseases attributable to LBW in 204 countries worldwide from 1990 to 2021. **(A)** Frontier analysis from 1990 to 2021. **(B)** Frontier analysis for 2021As illustrated in the figure. Black dots indicate the 15 countries with the greatest differences from the frontier line, red dots indicate the 5 countries with the most significant effective differences in high SDI regions, and blue dots indicate the 5 countries with the least effective differences in low SDI regions. SDI, socio-demographic index; ASDR, age-standardized disability-adjusted life year rate; LBW, low birth weight.

To evaluate a country's policy measures and interventions aimed at reducing the burden of neonatal diseases attributable to LBW based on its SDI level, we employed a frontier analysis model. This model plotted the frontier line according to the SDI values and the ASDR of various countries over a 32-year period (Figure 4A). The analysis revealed a general decrease in efficiency differences with increasing SDI levels (Figure 4B). Over the past 32 years, 197 countries or regions demonstrated a narrowing trend in efficiency disparities. In 2021, six countries—Andorra, Cuba, Israel, Niger, Somalia, and the Solomon Islands—achieved theoretical ASDR values. However, some countries, such as Pakistan, Sierra Leone, Ivory Coast, Nigeria, and Mali, exhibited significantly higher observed ASDRs compared to frontier values. Among high-SDI countries, the greatest efficiency differences were observed in the US Virgin Islands, the United States, Puerto Rico, Greenland, and Brunei. Conversely, certain low-SDI countries, including Somalia, Niger, Burkina Faso, Burundi, and the Solomon Islands, outperformed peers with similar SDI levels (Figure 4B, Supplementary Table S2).

## 4 Discussion

This study, based on a secondary analysis of GBD 2021 data, provides a comprehensive evaluation of the long-term trends in neonatal disease burden attributable to LBW across global, regional, and national scales. It quantifies national health inequalities across varying socio-demographic index (SDI) levels and assesses the efficiency and potential of different countries in reducing this disease burden. Over the past 32 years, the global burden of neonatal diseases attributable to LBW has steadily declined. However, the distribution of disease burden remains uneven, with an increasing concentration in lower-income countries over time. Despite these disparities, countries across all SDI levels exhibit potential for further reductions in the burden of neonatal diseases. These findings underscore the critical importance of strengthening preventive measures and optimizing the management of neonatal diseases attributable to LBW worldwide.

The study also reveals that the rate of decline in the global neonatal disease burden has accelerated significantly since 2012. This trend aligns with the global nutrition targets established by the World Health Assembly (WHA) in 2012, which aim to reduce LBW prevalence by 30% by 2025, and the “Zero Hunger” initiative proposed by the United Nations in 2021. These initiatives have driven substantial progress in reducing hunger, poverty, and improving maternal and child welfare globally (14, 20–22). Furthermore, the analysis highlights gender disparities, showing that male neonates experience a higher burden of diseases attributable to LBW compared to females. This disparity may be attributed to the faster growth rates of male fetuses (23), which demand higher nutrient intake, as well as their greater susceptibility to adverse pregnancy outcomes and neonatal diseases linked to air pollution. These findings emphasize the need for targeted strategies to address gender-specific vulnerabilities in neonatal health (24).

Previous studies have revealed significant variations in the burden of neonatal diseases across countries with differing levels of socio-demographic index (SDI) development. Research by Ou et al. (13) corroborates our findings, showing that this burden is predominantly concentrated in low and lower-middle SDI regions. This disparity is likely influenced by factors such as low educational attainment, cultural traditions that lead to younger maternal age (25, 26), inadequate nutritional guidance during pregnancy due to poverty and hunger (27), a high prevalence of gestational diseases such as hypertensive disorders (28, 29), and limited access to prenatal care, pediatric healthcare, and referral systems for sick newborns in countries with fragile healthcare infrastructures (30–32). Additionally, neonatal diseases linked to low birth weight (LBW) in these underdeveloped areas strain scarce healthcare resources, reducing accessibility to perinatal and neonatal care and perpetuating a vicious cycle of poor health outcomes.

The introduction of the millennium development goals and sustainable development goals has brought growing public attention to health inequality. Our study shows that while global socioeconomic improvements have led to a greater absolute reduction in disease burden among lower-SDI populations, relative inequality has widened, with the disease burden becoming increasingly concentrated among poorer populations. This aligns with prior research (33). Importantly, this trend is not confined to individual countries but reflects collective global dynamics. In 1990, low and lower-middle SDI countries accounted for 76.98% of the global population and 97.78% of total global DALYs lost. By 2021, these countries comprised 41.60% of the population but still represented 85.49% of total DALYs lost (Supplementary Table S3). Our findings further confirm that during efforts to achieve these global goals, health service interventions have expanded faster among the poorest fifth of the population compared to the richest fifth. However, coverage rates among the poorest remain significantly lower than those of the wealthiest (34–36), presenting a substantial challenge. The underlying drivers of this phenomenon include the inequitable distribution of socioeconomic resources and disparities in public health policies and interventions between countries. Addressing these issues should be a critical focus for the public health field.

Our frontier analysis highlights substantial progress in reducing the neonatal disease burden attributable to LBW, with 197 countries achieving significant improvements from 1990 to 2021. This underscores the effectiveness of intervention policies and measures implemented worldwide. However, as SDI levels rise, disparities in disease burden are becoming increasingly concentrated in lower-SDI countries, indicating that these regions hold substantial potential for further reductions through targeted policy adjustments and interventions. While frontier countries exist at all SDI levels, the most remarkable examples are in low-SDI settings. Despite limited socioeconomic resources, these countries have achieved the theoretical minimum disease burden for their developmental stage through efficient resource utilization, distinguishing themselves among peers. For instance, Somalia, despite long-term conflict and some of the world's poorest health indicators (37), has made consistent progress since the establishment of its federal government in 2012. In 2014, it introduced the Essential Package of Health Services (EPHS), which enhanced healthcare governance, institutional capacity, and sustainable health financing, increased per capita health expenditure, and reduced perinatal and child mortality rates (38). Additionally, with the support of UN agencies and humanitarian organizations, Somalia has implemented a four-tier healthcare system alongside the Somali Health and Nutrition Program (SHINE). These initiatives have significantly improved perinatal and neonatal care, leading to a reduction in neonatal mortality rates (38–40). Conversely, some high-SDI countries, such as the United States, exhibit disproportionately high neonatal mortality rates relative to their developmental potential. This disparity aligns with findings by Geronimus et al. (41), suggesting contributory factors such as the growing prevalence of elderly pregnancies and the increased incidence of iatrogenic premature births (42–44). Moreover, an imbalanced focus in health policies—compounded by inadequate attention to perinatal care in ethnic minority populations, shaped by a legacy of racial segregation and systemic discrimination—likely exacerbates the rising disease burden in the United States (41, 45). This divergence underscores a critical insight: improvements in health outcomes are not inherently tied to socioeconomic development. A broader array of factors can hinder progress, necessitating a dual approach. While advancing the successes of leading nations in neonatal care, it is equally vital to address barriers impeding progress in lagging nations.

Our analysis of trends and inequalities in the burden of neonatal diseases attributable to low birth weight (LBW) reveals crucial global patterns and underlying factors. These findings serve as valuable references for designing comprehensive perinatal and neonatal healthcare policies. Prioritizing medical assistance in low-socioeconomic countries is essential to improving the quality, accessibility, and equality of maternal and neonatal healthcare services, thereby reducing the burden of LBW-related neonatal diseases. Simultaneously, reversing health disparities associated with these diseases demands targeted interventions. For resource-constrained nations, optimizing healthcare resource allocation, implementing cost-effective interventions, addressing behavioral and environmental risk factors, promoting routine perinatal checkups, and enhancing neonatal screening and care services are pivotal steps. In large-population countries like China, the government has made significant progress over the past three decades. A robust prenatal registration and examination system has increased prenatal examination rates from 69.7% in 1992 to 97.6% in 2021, while hospital delivery rates have risen from 50.6% in 1990 to 99.9% in 2021 (46, 47). These efforts have significantly reduced age-standardized mortality and disability rates. However, the disease burden remains considerable due to China's vast population. Furthermore, a declining fertility rate has been accompanied by a steady rise in LBW incidence, from 2.9% in 2017 to 3.7% in 2021 (47). To avoid replicating the challenges observed in the U.S., the Chinese government must intensify its focus on reproductive health and optimal childbearing practices.

This study has several limitations. First, the GBD study defines LBW as below the TMREL (theoretical minimum risk exposure level), which is estimated using a model that incorporates gestational age. While this approach quantifies the effect of gestational age adjustments on LBW, it limits direct comparability with other studies. Second, GBD 2021 data demonstrate sparse heterogeneity, with incomplete statistical data for some countries. The use of mathematical modeling to fill these gaps may underestimate the burden of LBW-related neonatal diseases, especially in low-development countries. Third, while this study offers a macro-level assessment of the disease burden across global, regional, and national levels, it does not account for subnational disparities. Further research is needed to explore micro-level trends and associations within countries.

## 5 Conclusion

Over the past 32 years, the global burden of neonatal diseases associated with LBW has significantly decreased. However, disparities in the disease burden have worsened, highlighting persistent inequalities. To address this, the international community and governments must continue advancing the accessibility, equality, and quality of healthcare for pregnant women, mothers, and newborns.

## Data Availability

The datasets presented in this study can be found in online repositories. The names of the repository/repositories and accession number(s) can be found below: https://vizhub.healthdata.org/gbd-results/.
